# Prevalence and Characterization of *Staphylococcus aureus* and Methicillin-Resistant *Staphylococcus aureus* Isolated from Guangxi Dairy Farms

**DOI:** 10.3390/foods14132221

**Published:** 2025-06-24

**Authors:** Kai Ma, Jia Guo, Jie Hu, Qiuyuan Liu, Hui Wang, Ting Xue

**Affiliations:** 1 School of Life Sciences, Anhui Agricultural University, Hefei 230036, China; makaiunique@163.com (K.M.); 18037931607@163.com (J.G.); hujie4339@163.com (J.H.); 2 College of Veterinary Medicine, Anhui Agricultural University, Hefei 230036, China; liuqiuyuan95@163.com; 3 Joint Research Center for Food Nutrition and Health of IHM, Anhui Agricultural University, Hefei 230036, China

**Keywords:** *Staphylococcus aureus*, raw milk, *spa*, virulence

## Abstract

*Staphylococcus aureus* (*S. aureus*) is a major pathogen responsible for mastitis in dairy cows and can contaminate raw milk, thereby posing significant health risks to consumers. The emergence of methicillin-resistant *S. aureus* (MRSA) has further heightened public health concerns due to its antibiotic resistance and infectious potential. In this study, we examined the prevalence, virulence genes, antimicrobial resistance, *spa* types, and biofilm formation of *S. aureus* isolates from dairy farms in Guangxi Province, China. Among 242 randomly selected samples, 37 *S. aureus* strains were identified (15.3% infection rate), including 67.5% MRSA. Antibiotic resistance was observed in 78.4% of isolates, with 35.1% exhibiting multidrug resistance (MDR). Enterotoxin gene analysis showed *sea* as the most common (67.6%), followed by *ser* (54.1%) and *seh* (51.4%), whereas *seb* and *selj* were absent. All isolates formed biofilms in vitro, with 64.8% showing strong biofilm-forming ability. Staphylococcal protein A (*spa*) typing classified the 37 *S. aureus* strains into 11 *spa* types, with t030 being the most prevalent (43.2%). These findings indicate that *S. aureus* is moderately prevalent in raw milk, often carrying multiple virulence genes, forming robust biofilms, and showing antimicrobial resistance. The MRSA that is “latent” in raw milk reminds us of the need for monitoring at the farm level.

## 1. Introduction

As an opportunistic pathogen with zoonotic potential, *Staphylococcus aureus* (*S. aureus*) is capable of inducing a diverse range of infections, including superficial skin disorders and serious conditions, like osteomyelitis, endocarditis, and bloodstream infections [1,2]. It is a common cause of mastitis in dairy cows, which can transmit the pathogen to milk [3]. As dairy consumption rises, *S. aureus* contamination in milk poses significant risks to consumer health and public safety [4,5].

Antibiotics remain the main strategy for bacterial infection control. However, misuse of antimicrobial agents has led to multidrug-resistant *S. aureus*, which makes the treatment of *S. aureus* infection more challenging [6]. MRSA is one of the predominant prevalent antibiotic-resistant strains, which is resistant to methicillin and other β-lactam antibiotics [7]. In addition, the formation of biofilm is an important virulence factor in the pathogenic process of *S. aureus* and an important pathway for resisting external environmental stress [8]. *S. aureus* encodes various virulence genes, including toxic shock syndrome (TSS), hemolysin, and enterotoxins, etc. [9], among which enterotoxin is a common cause of staphylococcal food poisoning (SFP) [10]. Over 23 enterotoxins are known, categorized into SEs and Staphylococcal Enterotoxin-like (SEl), based on their ability to induce emesis. Classic SEs include *sea*, *seb*, and *see* genes, while SEl proteins are encoded by *selj* and *selp* [11,12].

Staphylococcal protein A (*spa*) contains a polymorphic X region with variable 24-bp repeats and the diversity of *spa* typing is caused by deletion of repeat units, repeats, and point mutations [13]. The *spa* typing of repeating sequences has higher discrimination than dose multilocus sequence typing. The *spa* typing method is a DNA sequence-based genotyping method that is relatively simple to implement [14]. Conserved regions flanking the X-region enable direct PCR amplification and sequencing. This approach provides reproducible, clear, and interpretable results for *S. aureus* typing [15]. It can provide an appropriate reference for epidemiological investigation or strain distribution in a region.

In this study, we investigated *S. aureus* in dairy farms in Guangxi Province, China, and aimed to understand more about the epidemiological characteristics of *S. aureus* and MRSA in dairy farms in Guangxi Province, China, through strain isolation, *spa* typing, antimicrobial susceptibility analysis, enterotoxin genes detection, and biofilm formation analysis.

## 2. Materials and Methods

### 2.1. Collection of Milk Samples

**Figure 1:**
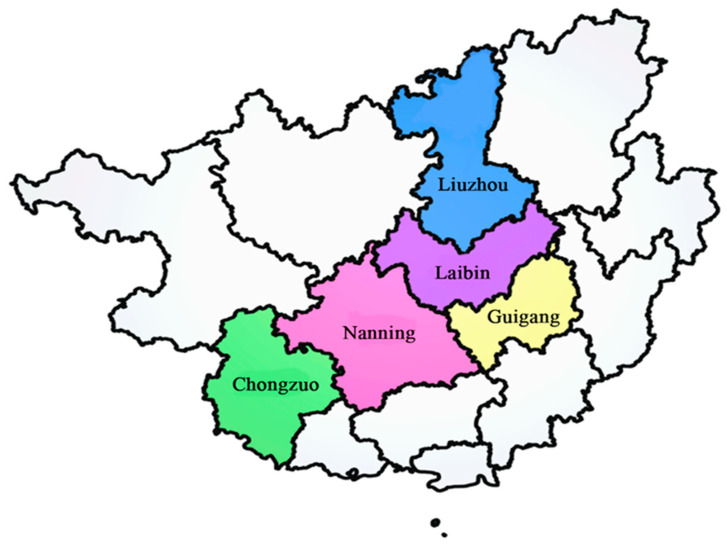
Map of sampling locations. The 5 regions are the main dairy-producing areas in Guangxi.

Between May 2024 and January 2025, 242 milk samples were collected from different dairy farms in Nanning, Laibin, Liuzhou, Guigang, and Chongzuo, Guangxi Province, China (Figure 1). Milk samples were collected from dairy farms using a random method. Sampling was carried out after the sampler had disinfected his/her hands with 75% alcohol. The samples were placed in sterile 50 mL centrifuge tubes and transported to the laboratory under low-temperature conditions for further analysis.

### 2.2. Identification and Isolation of S. aureus

The isolation and identification of *S. aureus* were conducted using a modified protocol from a previous study [16]. In short, milk samples were first enriched in tryptic soy broth (TSB; Difco) for 8 h, followed by transfer to TSB with 7.5% NaCl and incubated at 37 °C for 16 h with shaking. Then, the cultures were serially diluted, spread onto TSB agar plates, and incubated inverted at 37 °C for 16 h, and single colonies were selected and incubated overnight. The genomes of the suspected strains were extracted separately using a TIANamp Bacteria DNA Kit (TianGen Biotech, Beijing, China). The putative *S. aureus* isolate was identified by sequencing with the previously described 16S rDNA primers (Table 1). Verified *S. aureus* isolates were preserved at −80 °C in 30% glycerol for further analysis.

### 2.3. Identification of mecA and Enterotoxin Genes

All isolates were analyzed for the presence of the *mecA* gene and various staphylococcal enterotoxin (SE) genes using PCR, with primer sequences listed in Table 1. The PCR product was detected in a 1% agarose gel containing a nucleic acid dye and subsequently observed under UV illumination. The MRSA strain was identified by the detection of the *mecA* gene, while a panel of SE-associated genes—*sea*, *seb*, *see*, *seg*, *sec*, *sed*, *seh*, *sei*, *ser*, *selj*, and *selp*—were simultaneously screened to assess potential virulence [16].

### 2.4. Antimicrobial Susceptibility Testing

Antimicrobial susceptibility was assessed using the disk diffusion method according to Clinical and Laboratory Standards Institute (CLSI) guidelines [17]. Sensitivity to vancomycin and oxacillin was determined by broth microdilution following CLSI guidelines. Briefly, the strain to be tested was shaken and cultivated overnight; then, the bacterial solution was diluted to OD_600_ = 0.5, and 100 μL liquid was used to coat the plate. The drug-sensitive paper sheet was pasted onto the plate and placed in a 37 °C incubator for 18 h. Then, the circle of inhibition was measured. Strain resistance was evaluated according to the size of the ring of inhibition. The antimicrobial agents tested included oxacillin (OXA), erythromycin (ERM), ofloxacin (OFX), vancomycin (VAN), gentamicin (CN), kanamycin (KAN), tetracycline (TE), chloramphenicol (CM), ciprofloxacin (CIP), linezolid (LEZ), sulfamethoxazole-trimethoprim (SXT), and nitrofurantoin (NIT). *S. aureus* was resistant to three or more antibiotics, which was defined as a multidrug-resistant strain, and the quality control strains were *S. aureus* ATCC25923 and ATCC29213.

### 2.5. Biofilm Formation Assay

According to a previous description, the 96-well microtiter plate method was used for biofilm formation analysis [18]. Briefly, strains cultured overnight were diluted to OD_600_ = 0.03, transferred to 96-well microtiter plates, and incubated at 37 °C for 24 h. After incubation, the supernatant was discarded, and each well was gently rinsed three times with PBS and left to air dry. The adherent biofilms were stained using 0.2% crystal violet (CV) for 30 min, rinsed twice with PBS to remove excess CV, and then solubilized using 33% acetic acid. The optical density (OD) was determined at 492 nm using a microplate reader. The negative control (ODc): TSB OD and biofilm formation capacity were analyzed based on the OD_492_ absorbance value [17]: (i) no biofilm producer: OD ≤ ODc; (ii) weak: ODc < OD ≤ (2 × ODc); (iii) moderate: (2 × ODc) < OD ≤ (4 × ODc); and (iv) strong: (4 × ODc) < OD.

### 2.6. spa Typing

Referring to the reported literature, *spa* typing was performed on all isolated strains [19]. The isolated *S. aureus spa* gene polymorphic X region was amplified using primers spa-1113F and spa-1514R obtained from the Ridom Spa Server http://www.spaserver.ridom.de/ (accessed on 6 November 2024). The amplification products were purified, recovered, and sequenced, and the *spa* type was analyzed in this database.

### 2.7. Statistical Analysis

Each experiment was performed in triplicate. The results are expressed as the mean value accompanied by the standard deviation (SD). Statistical analyses were conducted using SPSS software (version 18.0; SPSS Inc., Chicago, IL, USA), and differences were considered statistically significant when *p* < 0.05.

## 3. Results

### 3.1. Isolation and Identification of S. aureus

**Figure 2:**
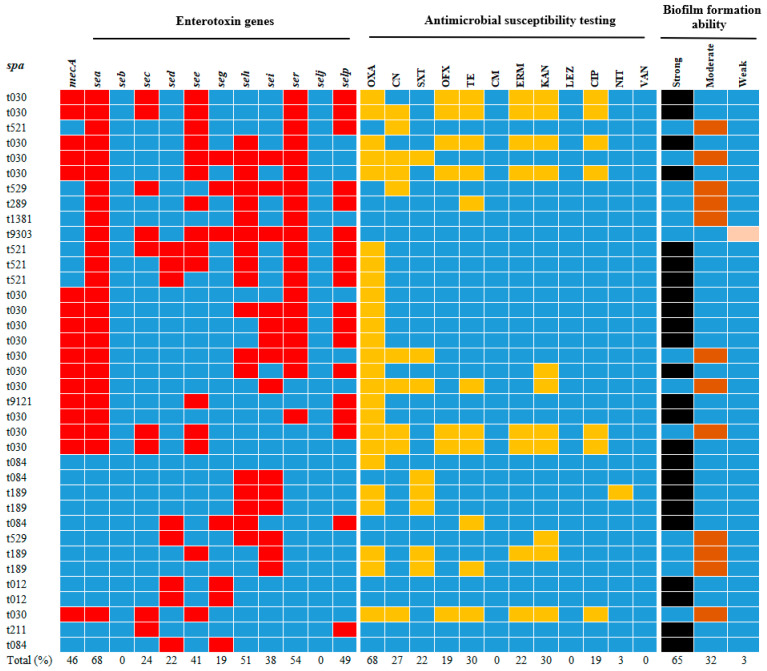
Enterotoxin genes, antimicrobial susceptibility test (AST), biofilm formation ability, *mecA* gene, and molecular characteristics of 37 *S. aureus* isolates from Guangxi Province. The 37 isolates were grouped into 11 *spa* types. Blue indicates a lack of the gene or no resistance; red indicates detection of the gene under study; yellow indicates resistance; and black, brown, and pink indicate strong biofilm formation capacity, medium biofilm formation capacity, and weak biofilm formation capacity, respectively. Oxacillin (OXA), erythromycin (ERM), ofloxacin (OFX), vancomycin (VAN), gentamicin (CN), kanamycin (KAN), tetracycline (TE), chloramphenicol (CM), ciprofloxacin (CIP), nitrofurantoin (NIT), sulfamethoxazole-trimethoprim (SXT), linezolid (LEZ).

A total of 37 *S. aureus* strains were isolated by16S rDNA gene sequencing from 242 samples collected from dairy farms in Guangxi Province, China, and including 9 strains from Liuzhou (9/70, 12.9%), 5 strains from Laibin (5/38, 13.2%), 14 strains from Guigang (14/66, 21.2%), 6 strains from Nanning (6/38, 15.8%), and 3 strains from Chongzuo (3/30, 10%). The overall detection rate of *S. aureus* was 15.3%, with 25 strains identified as methicillin-resistant strains and 17 strains harboring the *mecA* gene (Table 2, Figure 2).

### 3.2. Analysis and Identification of spa Typing of Isolates

**Figure 3:**
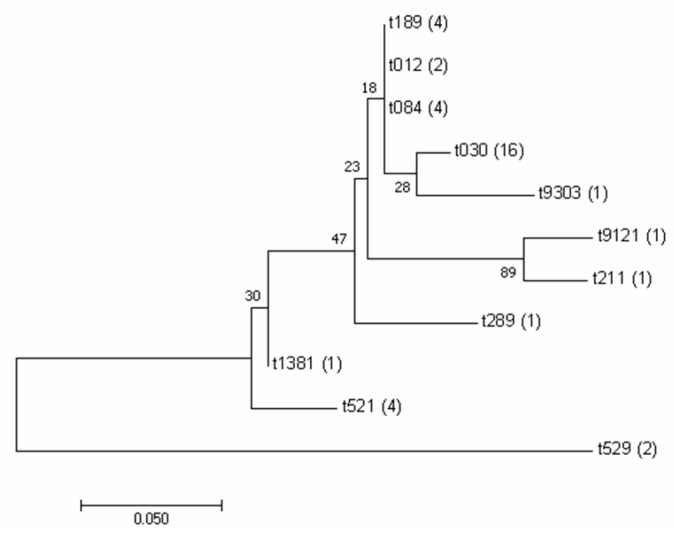
Phylogenic tree of the 37 isolates based on *spa* typing. The phylogenetic tree of 37 isolates was constructed by *spa* typing, and their genetic relationship was analyzed. The numbers in parentheses indicate the number of strains in the classification.

*spa* typing analysis of 37 *S. aureus* isolates identified 11 distinct *spa* types (Table 3). The most prevalent type was t030 (43.2%, 16/37), followed by t521, t084, and t189 (10.8%, 4/37 each), t529 and t012 (5.4%, 2/37 each), and t211, t289, t1381, t9303, and t9121 (1 strain each). The *spa* types of MRSA isolates were t030 and t9121. The 37 *S. aureus* isolates, representing 11 *spa* types, were clustered using MEGA 7.0 (Figure 3). 

### 3.3. Enterotoxin Gene Frequencies in Isolates

Enterotoxins contribute to SPF; thus, we analyzed the toxin gene profiles of all *S. aureus* isolates. A total of 9 enterotoxin genes were detected in 37 strains of *S. aureus*. The SEs with the highest frequency were *sea* (67.6%, 25/37), followed by *ser* (54.1%, 20/37), *seh* (51.4%, 19/37), *selp* (48.7%, 18/37), *see* (40.5%, 15/37), *sei* (37.8%, 14/37), *sec* (24.3%, 9/37), sed (21.6%, 8/37), and *seg* (18.9%, 7/37), and the enterotoxin genes *seb* and *selj* were not detected (Table 4).

### 3.4. Antimicrobial Resistance Analysis of Isolates

**Figure 4:**
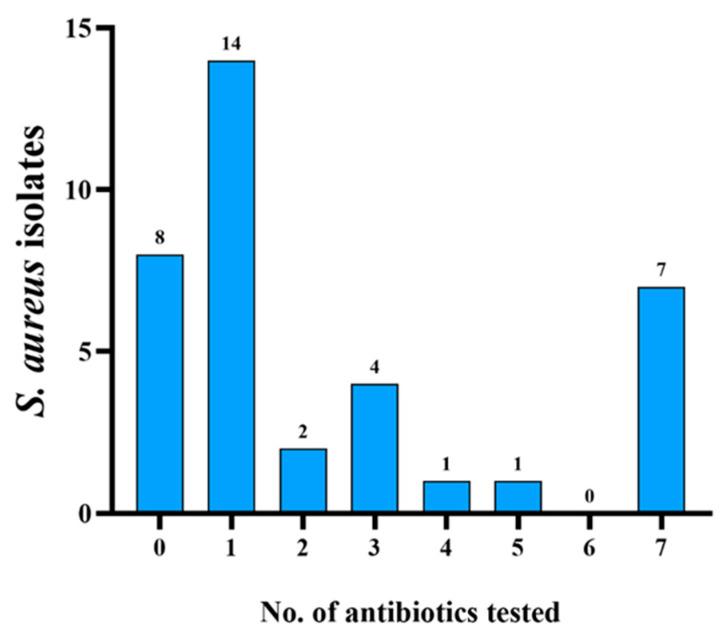
Frequency distribution of antimicrobial agent resistance patterns of *S. aureus*. The abscissa represents the number of antibiotics tested, and the ordinate represents the number of resistant strains. A total of 8 strains were susceptible to all antibiotics and 13 strains were resistant to three or more antibiotics.

The antimicrobial susceptibility profiles of 37 *S. aureus* isolates are shown in Table 5 and Figure 4. Of the *S. aureus* isolates, 8 strains (21.6%) were susceptible to all antibiotics, 14 strains (37.8%) were resistant to one antibiotic, 2 strains (5.4%) were resistant to both antibiotics, and 13 strains (35.1%) showed a multidrug-resistant phenotype (resistance to at least three antimicrobials). These isolates showed resistance to oxacillin (67.6%, 25/37), gentamicin (27%, 10/37), kanamycin (29.7%, 11/37), tetracycline (29.7%, 11/37), ofloxacin (18.9%, 7/37), sulfamethoxazole-trimethoprim (21.6%, 8/37), erythromycin (21.6%, 8/37), ciprofloxacin (18.9%, 7/37), and nitrofurantoin (2.7%, 1/37). All *S. aureus* isolates were susceptible to vancomycin, chloramphenicol, and linezolid.

### 3.5. Biofilm Formation Ability

**Figure 5:**
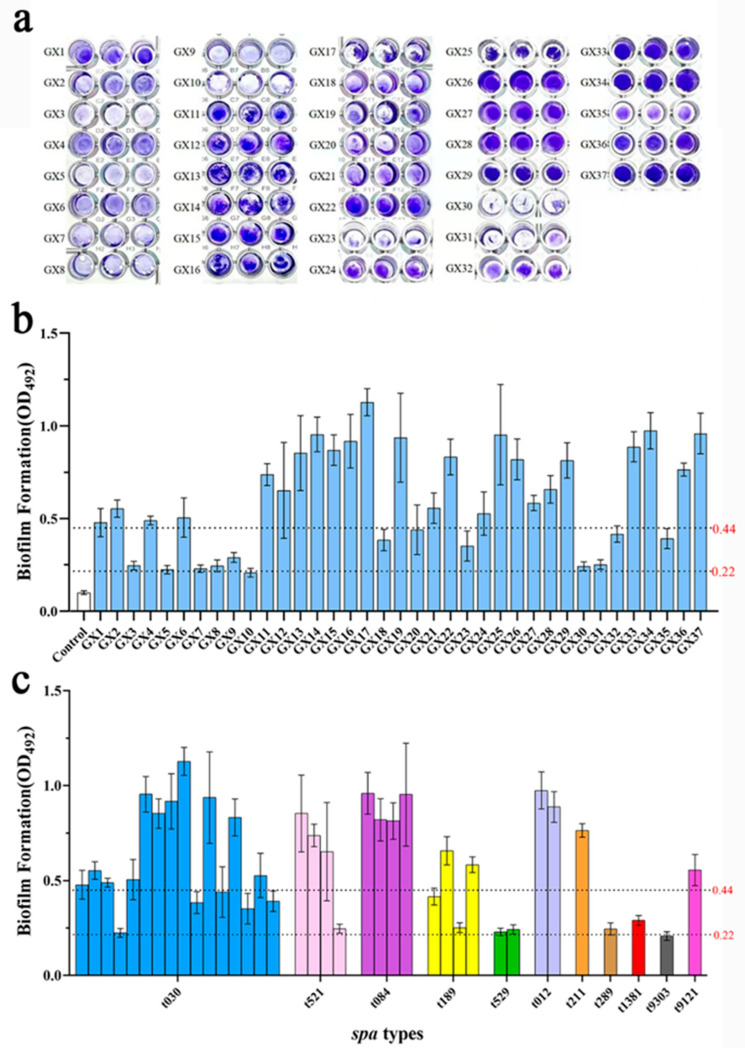
Biofilm formation ability of 37 *S. aureus* isolates. (**a**) A photograph of biofilm in 96-well plates after staining with crystal violet. (**b**) OD492 absorbance value of biofilm stained with crystal violet after dissolving in 33% glacial acetic acid. Values 0.22 and 0.44 indicate moderate biofilm-forming capacity and strong biofilm-forming capacity. (**c**) Different *spa* types and biofilm formation ability.

The biofilm-forming capacity of *S. aureus* is essential for stress resistance and virulence. This ability was evaluated using the crystal violet staining method. As shown in Figure 5a,b, all strains were able to form biofilms, of which 1 (2.7%, 1/37) strain showed weak biofilm formation ability, 12 (32.4%, 12/37) strains showed moderate biofilm formation ability, and 24 (64.8%, 24/37) strains showed strong biofilm formation ability. Among them, 11 strains of t030, 3 strains of t521, 4 strains of t084, 2 strains of t189, 2 strains of t012, 1 strain of t211, and 1 strain of t9121 had a strong biofilm-forming ability (Figure 5c). These results show that different *S aureus* isolated from dairy farms generally have the biofilm formation ability in vitro, which seriously endangers public health.

## 4. Discussion

*S. aureus* is a common foodborne pathogen. Previous studies have confirmed that *S. aureus* has been detected in raw milk, handmade yogurt, and other processed dairy products, posing public health risks [16,20,21]. Foodborne outbreaks caused by contaminated dairy products are the most obvious threat to public health posed by *S. aureus*, particularly those caused by strains with biofilm formation, enterotoxin production, and multidrug resistance [10]. For instance, one of the largest food poisoning outbreaks due to dairy Staphylococcal contamination occurred in Japan [22]. However, epidemiological data on *S. aureus* in dairy farms remain limited.

Here, we tested the contamination rate of raw milk from dairy farms in Guangxi Province, China, to assess the drug resistance, virulence, and biofilm-forming capacity of *S. aureus* isolates. In our research, conducted in raw milk from Guangxi, we found that 15.3% (37/242) of raw milk samples were positive for *S. aureus*. Our monitoring in Guangxi Province, China, dairy farms was comparable to the previously reported detection rate of *S. aureus* in raw milk in Iran (12.5%) [23], but lower than that reported in Malaysia (66.7%) [24] and Italy (41.0%) [25]. In contrast, our results showed a lower detection rate, indicating that the contamination of *S. aureus* was effectively controlled. These differences may be attributed to regional prevalence, variations in isolation test sensitivity, and hygiene conditions during milk production, transportation, and storage. However, it is important to note that our samples were primarily obtained from five regions in Guangxi, which may limit the representativeness of the findings. Additionally, the relatively small sample size may influence the accuracy of regional prevalence estimates. Therefore, expanding both the geographic scope and sample size in future studies will be essential for a more comprehensive assessment of the molecular epidemiology of *S. aureus* in the region. Furthermore, mastitis is a common disease in dairy cows that can lead to decreased milk yield and quality, higher culling rates, and substantial economic losses [26]. Infected cows are a major source of *S. aureus* contamination in milk, posing a threat to public health [27]. Hence, maintaining good hygiene practices and implementing strict preventative measures on dairy farms are crucial for reducing *S. aureus* contamination in both the environment and raw milk.

At present, the prevention and treatment of mastitis in dairy cows is mainly the use of antibiotics, driving the evolution of *S. aureus* resistance in animal populations. Therefore, antimicrobial susceptibility testing of isolates is necessary. Here, we found that 78.4% of the *S. aureus* isolates were resistant to at least one antibiotic, similar to previous studies where more than 90% of Chinese dairy cow isolates were resistant [28]. Most isolates are susceptible to nitrofurantoin, probably because this antibiotic is used less frequently, and our data show lower erythromycin resistance compared to other parts of China [17]. Similar to previous studies, 29.7% of *S. aureus* was resistant to kanamycin and tetracycline [29]. In recent years, MRSA has gained increasing awareness on farms and is easily transmitted to milk and humans who come into contact with it [30]. Ongoing monitoring of raw milk may help to prevent or mitigate the spread of MRSA strains through the dairy food chain, thereby reducing or avoiding the incidence of food poisoning caused by *S. aureus*. According to the article, all oxacillin MICs ≥ 2 mg/L were identified as MRSA [31]. Our results revealed that 35.1% of isolates were resistant to three or more antibiotics, and 67.6% (25/37) were identified as methicillin resistant. Resistance genes are the main cause of bacterial drug resistance. The *mecA* gene is the most common resistance gene in MRSA; thus, we tested the isolates for the *mecA* gene. However, only 45.9% (17/37) carried the *mecA* gene, differing from previous studies in which all methicillin-resistant strains harbored *mecA* [32]. The emergence of this phenomenon suggests that there may be new drug resistance genes. For example, Becker et al. found a plasmid carrying the *mecB* gene in 2018 during MRSA testing of isolates of *S. aureus*, leading to the emergence of methicillin resistance in the strain [33]. Meanwhile, the continuous identification of *mecA* homologs and intra- and interspecies genetic recombination of *mecA* and *mecC* promote the development of drug resistance in *S. aureus* [34]. Therefore, it is important to standardize the use of drugs to reduce the evolution of strains and to allow the development of targeted drugs against resistance genes. In this study, the increased prevalence of MRSA may be attributed to antibiotic overuse and inadequate environmental disinfection. Therefore, continuous monitoring is essential for controlling MRSA transmission in dairy products, preventing future contamination outbreaks, and mitigating risks to consumers.

*S. aureus* produces enterotoxins, which can cause food poisoning and damage the human body if contaminated milk is ingested. SFP is a common bacterial illness, and its occurrence has been linked to the expression of SE genes [35]. Twenty-two different enterotoxin genes have been reported since the first discovery of *sea* and *seb* from 1959 to 1960 [10], and although raw milk is sterilized, there may still be *S. aureus* that can survive the intervention [36]. Outbreaks caused by *S. aureus* enterotoxins have occurred during this period, posing a threat to the health of consumers. For example, there has been an outbreak of staphylococcal food poisoning in Japan, which is mainly caused by the clonal complex 81 (CC81) lineage and tested positive for *sea*, *seb*, and *seh* [37]. Therefore, we used PCR technology to detect the enterotoxin genes of 37 *S. aureus* strains. Studies have shown that the *sea* gene has the highest detection rate, consistent with previous findings indicating that *sea* is the predominant enterotoxin gene in clinical food poisoning cases in China and in isolates from raw milk [16,38]. In contrast, other studies have reported sec or sed as the most commonly detected enterotoxin genes in *S. aureus* isolates from raw milk [19,39]. The prevalence of these SEs in *S. aureus* may be influenced by regional variation and differences in sample sources. However, genes encoding novel SEs are widely present in *S. aureus*, and their role in pathogenesis may be underestimated.

*spa* typing is a commonly used method for *S. aureus* typing, and the common *spa* type in this study was t030, which is different from the results of other researchers, such as type t127 in *S. aureus* in Greece, and type t011 is the most common type in MRSA isolates of bovine origin [40,41], which may be due to the limited data on *spa* type distribution available for isolates. A previous study attempted to establish a relationship between *spa* type and biofilm formation capacity [42]. Biofilm formation is a key bacterial strategy for stress resistance and is considered a major virulence factor. Therefore, all *S. aureus* isolates were assessed for their biofilm formation capacity. The results indicate that while all isolates formed biofilms in vitro, no clear association with *spa* type was observed. *spa* typing is considered an effective genotyping tool for both national and international epidemiological surveillance due to its simplicity, reproducibility, and ease of result comparison [43]. However, the association between *spa* evolutionary lineages and phenotypic traits, such as antimicrobial resistance and virulence, remains poorly understood. In our study, all t030-type strains exhibited methicillin resistance, suggesting a possible association between the *spa* type and antibiotic resistance. Conversely, no clear link was observed between the *spa* type and the presence of enterotoxin genes. Among t030 strains, the number of enterotoxin genes detected ranged from 2 to 8. Although the limited sample size may constrain the generalizability of our findings, the potential relationship between *spa* genotypes and virulence characteristics provides valuable insight for the prevention and control of *S. aureus* contamination.

## 5. Conclusions

Our findings reveal that *S. aureus* isolates from raw milk in dairy farms in Guangxi Province, China, frequently harbor enterotoxin genes and exhibit biofilm-forming capacity, both of which are implicated in its pathogenesis. This suggests that these isolates are potentially contagious and may act as a trigger for foodborne infections. It is important to note that the high prevalence of MDR *S. aureus* make the treatment of *S. aureus* infection extremely challenging. Therefore, continuous monitoring of *S. aureus* resistance patterns in dairy products is crucial. At the same time, the 37 isolates were classified into 11 *spa* types, reflecting the genetic diversity of *S. aureus* in the region. Therefore, monitoring the infection status and molecular epidemiology of *S. aureus* in raw milk is crucial for developing effective control strategies to mitigate its transmission.

## Figures and Tables

**Table 1:** Oligonucleotide primers used in this study.

Gene	Primer	Primer Sequence (5′-3′)	Reference or Source
16S	27F	AGAGTTTGATCCTGGCTCAG	This study
	1492R	TACCTTGTTACGACTT	
*mecA*	mecA-F	GTTGTAGTTGTCGGGTTT	This study
	mecA-R	CCACATTGTTTCGGTCTA	
*spa*	spa-1113F	TAAAGACGATCCTTCGGTGAGC	Ridom
	spa-1514R	CAGCAGTAGTGCCGTTTGCTT	Ridom
*sea*	sea-F	GGTTATCAATGTGCGGGTGG	[16]
	sea-R	CGGCACTTTTTTCTCTTCGG	
*seb*	seb-F	GTATGGTGGTGTAACTGAGC	[16]
	seb-R	CCAAATAGTGACGAGTTAGG	
*sec*	sec-F	AGATGAAGTAGTTGATGTGTATGG	[16]
	sec-R	CACACTTTTAGAATCAACCG	
*sed*	sed-F	CCAATAATAGGAGAAAATAAAAG	[16]
	sed-R	ATTGGTATTTTTTTTCGTTC	
*see*	see-F	TGTATGTATGGAGGTGTAAC	[16]
	see-R	GCCAAAGCTGTCTGAG	
*seg*	seg-F	GTTAGAGGAGGTTTTATG	[16]
	seg-R	TTCCTTCAACAGGTGGAGA	
*seh*	seh-F	CAACTGCTGATTTAGCTCAG	[16]
	seh-R	CCCAAACATTAGCACCA	
*sei*	sei-F	GGCCACTTTATCAGGACA	[16]
	sei-R	AACTTACAGGCAGTCCA	
*ser*	ser-F	AGATGTGTTTGGAATACCCTAT	[16]
	ser-R	CTATCAGCTGTGGAGTGCAT	
*selj*	selj-F	GTTCTGGTGGTAAACCA	[16]
	selj-R	GCGGAACAACAGTTCTGA	
*selp*	selp-F	TCAAAAGACACCGCCAA	[16]
	selp-R	ATTGTCCTTGAGCACCA	

**Table 2:** Prevalence of *S. aureus* in milk from Guangxi Province, China.

Locations	No. of Samples	No. of *S. aureus* (%)	No. of MRSA * (%)
Liuzhou	70	9 (12.9%)	9 (12.9%)
Laibin	38	5 (13.2%)	3 (7.8%)
Guigang	66	14 (21.2%)	8 (12.1%)
Nanning	38	6 (15.8%)	3 (7.9%)
Chongzuo	30	3 (10%)	2 (6.6%)
Total	242	37(15.3%)	25

* MRSA: methicillin-resistant *S. aureus*.

**Table 3:** *spa* types of the isolated *S. aureus*.

*spa* Type	*spa* Repeat Succession	No. and Proportion of Isolates
t030	15-12-16-02-24-24	16 (43.2%)
t521	07-23-12-21-17-34-34-34-34-33-34	4 (10.8%)
t084	07-23-12-34-34-12-12-23-02-12-23	4 (10.8%)
t189	07-23-12-21-17-34	4 (10.8%)
t529	04-34	2 (5.4%)
t012	15-12-16-02-16-02-25-17-24-24	2 (5.4%)
t211	11-19-12-12-21-17-34-24-34-22-25	1 (2.7%)
t289	26-23-21-17-34-12-23-02-12-23	1 (2.7%)
t1381	07-23-21-16-34-33-34	1 (2.7%)
t9303	04-20-24-17	1 (2.7%)
t9121	11	1 (2.7%)

**Table 4:** Occurrence of enterotoxin genes of *S. aureus*.

Enterotoxin Genes	No. of *S. aureus*	Detection Rate
*sea*	25	67.6%
*seb*	0	0
*sec*	9	24.3%
*sed*	8	21.6%
*see*	15	40.5%
*seg*	7	18.9%
*seh*	19	51.4%
*sei*	14	37.8%
*ser*	20	54.1%
*selj*	0	0
*selp*	18	48.7%

**Table 5:** Number and percentage of antimicrobial-resistant *S. aureus* isolates.

Antibiotic Class	Antibiotic	No. of Resistant *S. aureus* (%)
β-Lactams	Oxacillin	25 (67.6%)
Aminoglycosides	Gentamicin	10 (27%)
	Kanamycin	11 (29.7%)
Tetracyclines	Tetracycline	11 (29.7%)
Sulfonamides	Sulfamethoxazole-trimethoprim	8 (21.6%)
Chloramphenicol	Chloramphenicol	0 (0%)
Macrolides	Erythromycin	8 (21.6%)
Quinolones	Ofloxacin	7 (18.9%)
	Ciprofloxacin	7 (18.9%)
Oxazolidinones	linezolid	0 (0%)
Nitrofurans	Nitrofurantoin	1 (2.7%)
Glycopeptide	Vancomycin	0 (0%)

## Data Availability

The original contributions presented in the study are included in the article, further inquiries can be directed to the corresponding authors.

## Abbreviations

The following abbreviations are used in this manuscript:

MIC	minimum inhibitory concentration
DNA	deoxyribonucleic acid
MRSA	methicillin-resistant *Staphylococcus aureus*
MDR	multidrug resistance
OXA	oxacillin
ERM	erythromycin
OFX	ofloxacin
VAN	vancomycin
CN	gentamicin
KAN	kanamycin
TE	tetracycline
CM	chloramphenicol
CIP	ciprofloxacin
NIT	nitrofurantoin
SXT	sulfamethoxazole-trimethoprim
LEZ	linezolid
